# An updated PREDICT breast cancer prognostication and treatment benefit prediction model with independent validation

**DOI:** 10.1186/s13058-017-0852-3

**Published:** 2017-05-22

**Authors:** Francisco J. Candido dos Reis, Gordon C. Wishart, Ed M. Dicks, David Greenberg, Jem Rashbass, Marjanka K. Schmidt, Alexandra J. van den Broek, Ian O. Ellis, Andrew Green, Emad Rakha, Tom Maishman, Diana M. Eccles, Paul D. P. Pharoah

**Affiliations:** 1 Department of Gynaecology and Obstetrics, Ribeirao Preto Medical School, University of Sao Paulo, Sao Paulo, Brazil; 2 Faculty of Medical Science, Anglia Ruskin University, Cambridge, UK; 3 Department of Oncology, University of Cambridge, Cambridge, UK; 4 National Cancer Registration and Analysis Service, Public Health England, London, UK; 5 Division of Molecular Pathology, Netherlands Cancer Institute, Amsterdam, The Netherlands; 6 Division of Psychosocial Research and Epidemiology, Netherlands Cancer Institute, Amsterdam, The Netherlands; 7 Division of Cancer and Stem Cells, School of Medicine, University of Nottingham and Nottingham University Hospitals NHS Trust, City Hospital, Nottingham, UK; 8 Cancer Sciences Academic Unit and Southampton Clinical Trials Unit, Faculty of Medicine, University of Southampton and University Hospital Southampton Foundation Trust, Southampton, UK

**Keywords:** Breast cancer, Prognosis

## Abstract

**Background:**

PREDICT is a breast cancer prognostic and treatment benefit model implemented online. The overall fit of the model has been good in multiple independent case series, but PREDICT has been shown to underestimate breast cancer specific mortality in women diagnosed under the age of 40. Another limitation is the use of discrete categories for tumour size and node status resulting in ‘step’ changes in risk estimates on moving between categories. We have refitted the PREDICT prognostic model using the original cohort of cases from East Anglia with updated survival time in order to take into account age at diagnosis and to smooth out the survival function for tumour size and node status.

**Methods:**

Multivariable Cox regression models were used to fit separate models for ER negative and ER positive disease. Continuous variables were fitted using fractional polynomials and a smoothed baseline hazard was obtained by regressing the baseline cumulative hazard for each patients against time using fractional polynomials. The fit of the prognostic models were then tested in three independent data sets that had also been used to validate the original version of PREDICT.

**Results:**

In the model fitting data, after adjusting for other prognostic variables, there is an increase in risk of breast cancer specific mortality in younger and older patients with ER positive disease, with a substantial increase in risk for women diagnosed before the age of 35. In ER negative disease the risk increases slightly with age. The association between breast cancer specific mortality and both tumour size and number of positive nodes was non-linear with a more marked increase in risk with increasing size and increasing number of nodes in ER positive disease.

The overall calibration and discrimination of the new version of PREDICT (v2) was good and comparable to that of the previous version in both model development and validation data sets. However, the calibration of v2 improved over v1 in patients diagnosed under the age of 40.

**Conclusions:**

The PREDICT v2 is an improved prognostication and treatment benefit model compared with v1. The online version should continue to aid clinical decision making in women with early breast cancer.

**Electronic supplementary material:**

The online version of this article (doi:10.1186/s13058-017-0852-3) contains supplementary material, which is available to authorized users.

## Background

**Fig. 1:**
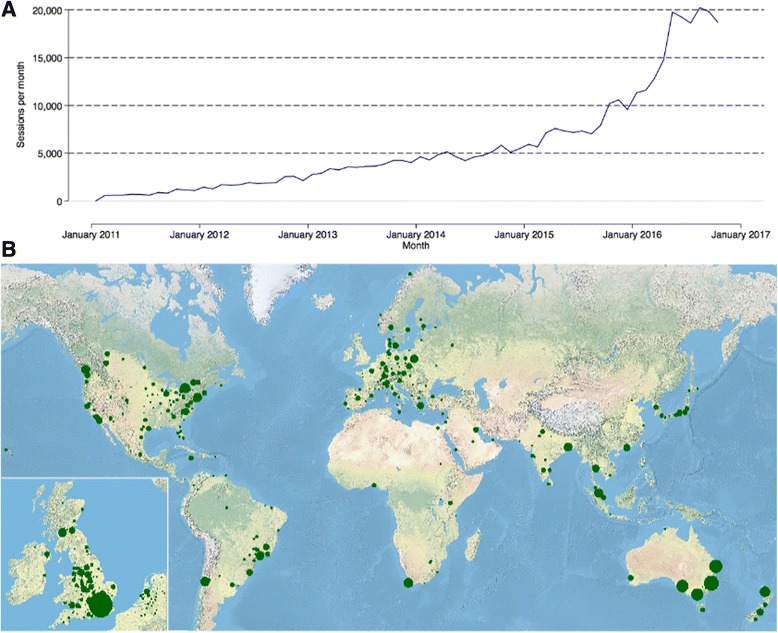
Web access to the online version of PREDICT at www.predict.nhs.uk, January 2011–October 2016. **a** Access per month. **b** Access by city. *Source*: Google Analytics (Mountain View, CA, USA)

The PREDICT breast cancer prognostication and treatment benefit prediction model (v1) was developed in 2010 [1] using data from the East Anglia Cancer Registration and Information Centre (ECRIC) for model fitting and data from the West Midlands Cancer Intelligence Unit for model validation [1]. PREDICT was implemented as a web-based tool for clinicians in January 2011 (www.predict.nhs.uk), and since then the use of the tool has increased steadily. In October 2016, the website was accessed over 20,000 times (Fig. 1a) from locations all over the world (Fig. 1b). The model is endorsed by the American Joint Committee on Cancer having met its criteria for inclusion of risk models for individualized prognosis in the practice of precision medicine [2] and is the only breast cancer prognostic model currently available online that has been endorsed by the American Joint Committee on Cancer [3].

The original model used tumour size in five categories (1–9 mm, 10–19 mm, 20–29 mm, 30–49 mm and 50 mm+), node status in five categories (0, 1, 2–4, 5–9 and 10+ positive nodes), tumour grade (1–3), oestrogen receptor (ER) status and mode of detection (clinical/screening) to estimate breast cancer-specific mortality at 5 and 10 years, as well as age to estimate non-breast cancer mortality at 5 and 10 years. The predicted benefit of adjuvant chemotherapy classified as first-, second- or third-generation and adjuvant hormone therapy was taken from the meta-analyses of the Early Breast Cancer Trialists Collaborative Group [4]. The model was subsequently validated in independent case series from British Columbia [5], The Netherlands [6, 7] and Malaysia [8], as well as two additional case series from the United Kingdom (the Prospective study of Outcomes in Sporadic and Hereditary breast cancer [POSH] study [9] and the Nottingham Breast Cancer Study [10]). Human epidermal growth factor receptor 2 (HER2) status (v1.2) and Ki67 status (v1.3) were also incorporated into the model, resulting in small improvements in discrimination of the model [10, 11].

Although the overall fit of the model has been good in multiple independent case series, PREDICT has been shown to underestimate breast cancer-specific mortality in women diagnosed under the age of 40 years, particularly those with ER-positive disease [9]. Another limitation of the model is the use of discrete categories for tumour size and node status, which result in ‘step’ changes in risk estimates on moving from one category to the next. For example, a woman with an 18-mm or 19-mm tumour will be predicted to have the same breast cancer-specific mortality if all the other prognostic factors are the same, whereas breast cancer-specific mortality for a 19-mm tumour will differ from that of women with a 20-mm tumour. We have therefore re-fitted the PREDICT prognostic model using the original cohort of cases from East Anglia with updated survival time to take into account age at diagnosis and to smooth out the survival function for tumour size and node status. The fit of the model has been tested in three independent data sets that have also been used to validate the original version of PREDICT.

## Methods

### Patient data

#### Model development data

The primary analysis was based on data from patients with invasive breast cancer diagnosed in East Anglia, UK, between 1999 and 2003 identified by ECRIC. ECRIC covered a catchment area population of approximately 5.5 million people and registers all malignant tumours occurring in people resident in East Anglia at the time of diagnosis. ECRIC also recorded prospectively demographic, pathologic, staging, general treatment and outcome information. Death certificate flagging through the Office for National Statistics provides the registries with notification of deaths. The lag times for this are a few weeks for cancer deaths and 2 months to 1 year for non-cancer deaths. In addition, ECRIC staff checked vital status by querying the National Health Service Strategic Tracing Service. Vital status was ascertained at the end of June 2013, and all analyses were censored on 31 December 2012 to allow for delay in reporting of vital status. Breast cancer-specific mortality was defined as deaths where breast cancer was listed as the cause of death on part 1a, 1b or 1c of the death certificate.

Information obtained from ECRIC included age at diagnosis, number of lymph nodes sampled and number of lymph nodes positive, tumour size, histological grade, ER status, mode of detection (screening vs. clinical), information on local therapy (wide local excision, mastectomy, radiotherapy), and type of adjuvant systemic therapy (chemotherapy, endocrine therapy, both). Exact chemotherapy regimens are unknown, but the majority of patients with breast cancer in the ECRIC population received first- or second-generation chemotherapy during this time period. Patients who did not undergo surgery, patients with incomplete local therapy (wide local excision without radiotherapy) and patients with fewer than four nodes excised with a diagnosis of node-negative disease were excluded from the analyses, leaving a study population of 5738 individuals. Of these 1977 (34%) had less than 10 years of potential follow-up.

#### *Validation samples*

From the Nottingham/Tenovus Breast Cancer Study (NTBCS), data were available for 2232 cases of invasive breast cancer treated in Nottingham from 1989 to 1998. Of these, 506 node-negative cases were excluded because of inadequate axillary node staging (fewer than four nodes sampled), leaving 1726 patients (ER-negative, *n* = 452; ER-positive, *n* = 1274) for the validation study. Outcome data were obtained on a prospective basis. Patients were followed at 3-month intervals initially, then at 6-month intervals, and then annually for a median period of 111 months (range 4–211 months). At death, the hospital notes are examined and deaths allocated to with/from breast cancer or to without known breast cancer. For those who were lost to follow-up, hospital notes were retrieved and checked. Vital status was ascertained at the end of October 2012. Breast cancer-specific mortality was defined as deaths where breast cancer was listed as the cause of death on part 1a, 1b or 1c of the death certificate. Breast cancer-specific survival was defined as the interval between the operation and death resulting from breast cancer, death being scored as an event, and patients who died as a result of other causes or were still alive were censored at the time of last follow-up.

For the Breast Cancer Outcome Study of Mutation Carriers (BCOS), data collection has been described previously [7]. In short, we used data from a hospital-based cohort of consecutive females diagnosed at <50 years of age with invasive breast cancer, identified through medical registries of participating hospitals or the Netherlands Cancer Registry. Patients diagnosed between 1990 and 2000 with unilateral stages I–III breast cancer without a previous cancer diagnosis (except non-melanoma skin cancer), for whom complete data on tumour size, nodal status, receipt of adjuvant systemic therapy, and follow-up were available, were included. Information about diagnosis and treatment (e.g., histological tumour grade, stage, adjuvant chemotherapy and endocrine systemic treatment; before about 2004 no trastuzumab was administered), ER and progesterone receptor status, HER2 and angiolymphatic invasion were gathered from original pathology reports and/or determined using reviews of whole slides and staining of tumours in tissue microarrays. Follow-up data were obtained from the medical registries from the participating hospitals and/or linkage with the Dutch municipal registry through the Netherlands Cancer Registry (last follow-up update in 2013).

The Prospective Study of Outcomes in Sporadic and Hereditary Breast Cancer (POSH) is a multicentre prospective observational cohort study of 2609 young women diagnosed with breast cancer in the United Kingdom between 2000 and 2008 [12]. Information obtained in the POSH cohort included age at diagnosis, histological grade, tumour size, number of positive lymph nodes, ER status, adjuvant chemotherapy, chemotherapy regimen and adjuvant hormone therapy. Outcome data were obtained through flagging with the Office for National Statistics. Vital status was ascertained at the end of June 2015, and all analyses were censored on 31 December 2014 to allow for delay in reporting of vital status. Breast cancer-specific mortality was defined as deaths where breast cancer was listed as the cause of death on part 1a, 1b or 1c of the death certificate. A total of 1374 of the participants (53%) had less than 10 years of potential follow-up. The validation studies were approved by the relevant research ethics committees, and all participants provided written informed consent.

#### Statistical methods

Separate models were derived for ER-positive and ER-negative breast cancer. The models were derived using Cox regression to estimate the coefficients associated with each risk factor. The non-linear risk relationships between continuous variables (age, tumour size and number of positive nodes) and breast cancer death were modelled using multivariable fractional polynomials [13]. The variables for the final models were selected by sequential backward elimination [14]. The effects of adjuvant chemotherapy and adjuvant hormone therapy were constrained to the effects reported for standard anthracycline-based chemotherapy and adjuvant tamoxifen from an updated analysis of the Early Breast Cancer Trialists Collaborative Group [15]. After fitting of the models, smoothed functions for baseline hazard of breast cancer-specific mortality were derived as follows. First, the baseline cumulative hazard was estimated for each patient. Then the logarithmic value of the baseline hazard was regressed against time using a univariate fractional polynomial function. The resulting functions were used to estimate the cumulative baseline hazard at 10 years.

A similar approach was used to model non-breast cancer mortality using Cox regression and multivariable fractional polynomials to obtain a function for other mortality with age. The smoothed baseline hazard function for non-breast-specific mortality was derived as described above.

### Calculation of predicted mortality for validation sample

A prognostic index (PI) for each patient was calculated as the sum of the weighted prognostic factors where the weights were the β-coefficients from the Cox regression and the logarithmic HRs for the effects of adjuvant chemotherapy and hormone therapy from clinical trials. A non-breast cancer mortality index (MI) was calculated as the weighted prognostic factor for non-breast cancer mortality. The absolute risk of breast cancer death (H_B_) before time *t*, assuming no competing mortality, is estimated by the following formula:

$$ {\mathrm{H}}_{\mathrm{B}} = 1\ \hbox{-}\ \exp \left(\hbox{-} \exp \left(\mathrm{PI}\right)*{\mathrm{B}\mathrm{Sb}}_t\right) $$

and the equivalent formula for the cumulative risk of non-breast cancer mortality (H_O_) is:

$$ {\mathrm{H}}_{\mathrm{O}} = 1\ \hbox{-}\ \exp \left(\hbox{-} \exp \left(\mathrm{MI}\right)*{\mathrm{BSo}}_t\right) $$

where BSb_*t*_ is the cumulative baseline hazard for breast cancer mortality at time *t* and BSo_*t*_ is the cumulative baseline hazard for non-breast cancer mortality at time *t*.

These are competing risks, so the cumulative risk of breast cancer mortality at time *t* is

$$ \mathrm{R}\mathrm{b} = {\mathrm{H}}_{\mathrm{B}}*\left({1\ \hbox{--}\ \mathrm{H}}_{\mathrm{O}}\right) $$

and the cumulative risk of non-breast cancer mortality is

$$ \mathrm{R}\mathrm{o} = {\mathrm{H}}_{\mathrm{O}}*\left({1\ \hbox{--}\ \mathrm{H}}_{\mathrm{B}}\right) $$

We also estimated the 10-year predicted breast cancer-specific mortality and other mortality using the current online version of PREDICT (v1.3).

Model calibration was analysed as a comparison of the predicted mortality estimates from each model with the observed mortality. In addition to comparing calibration in the complete data set, we evaluated calibration within strata of other prognostic variables. We also evaluated calibration within quintiles of predicted mortality. A goodness-of-fit test was carried out by using a χ^2^ test based on the observed and predicted number of events within each quintile (5 *df*). Model discrimination was evaluated by calculating the AUC calculated for 10-year mortality. This is a measure of how well each version of the model identifies those patients with worse survival. The AUC is the probability that the predicted mortality of a randomly selected patient who died will be higher than the predicted mortality of a randomly selected survivor. Comparison between the new model and v1 was made using the method of DeLong [16]. A goodness-of-fit test was carried out by using a χ^2^ test based on the observed and predicted number of events in quintiles of predicted risk (5 *df*). All analyses were carried out using Stata version 14 software (StataCorp, College Station, TX, USA).

## Results

The model fitting was carried out using data for 1020 women with ER-negative disease, 333 of whom had died as a result of breast cancer and 107 of whom had died as a result of other causes within 10 years of follow-up, as well as data for 4718 women with ER-positive breast cancer, 599 of whom had died as a result of breast cancer and 511 of whom had died as a result of other causes within 10 years of follow-up. Tumour size, number of positive nodes and tumour grade were significant prognostic factors for ER-negative disease in the Cox regression implemented within a multivariable fractional polynomial model. For ER-positive disease, age at diagnosis, tumour size, number of positive nodes, tumour grade and mode of detection were significant. The fractional polynomial functions and associated logarithmic HRs are shown in Table 1.

**Table 1:** Fractional polynomial functions and associated logarithmic HRs for age at diagnosis, tumour size, number of positive nodes, tumour grade and mode of detection by oestrogen receptor status

Prognostic factor	Function	Log HR	*P* value
ER-negative breast cancer specific mortality			
Age	= age − 56.325	0.00894	0.025
Tumour size, mm	= (size/100)^1/2^ − 0.5090	2.109	<0.0001
Number of positive nodes	=1/[(nodes + 1)/10] ^1/2^ − 1.72	−0.705	<0.0001
Grade	= grade	0.259	0.028
ER-positive breast cancer-specific mortality			
Age 1	= (age/10)^−2^ − 0.0287	34.53	0.001
Age 2	= (age/10)^−2^ × ln(age/10) − 0.0510	−34.20	0.001
Tumour size, mm	= ln(size/100) + 1.5452	0.7531	<0.0001
Number of positive nodes	= ln((nodes + 1)/10) + 1.3876	0.7069	<0.0001
Grade	= grade	0.7467	<0.0001
Screen-detected	screen detected = 1	−0.2763	0.016
Non-breast cancer mortality			
Age	= (age/10)^2^ − 34.234	0.0698	<0.0001

*ER* Oestrogen receptor

**Fig. 2:**
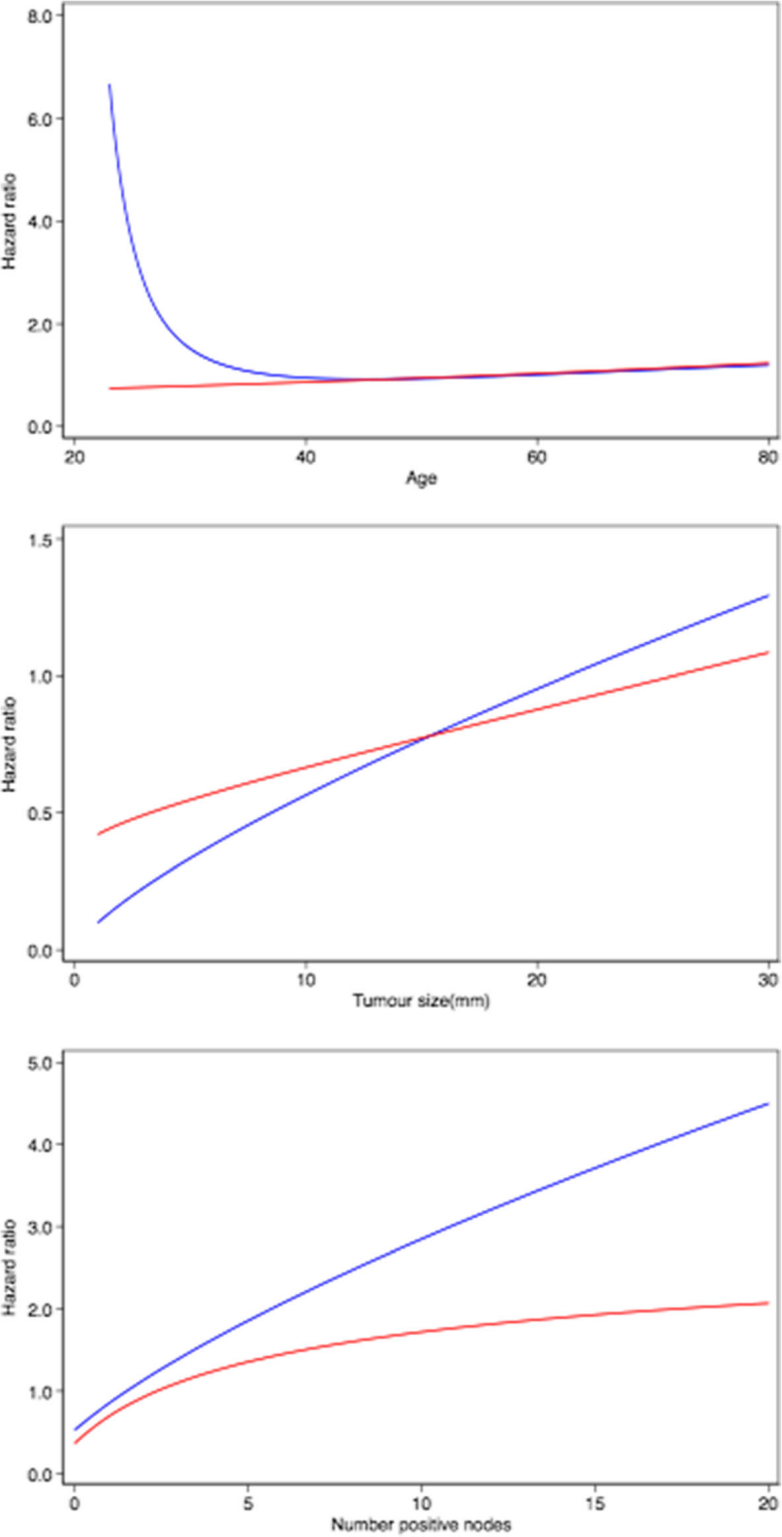
Breast cancer-specific mortality HR functions for age, tumour size and number of positive nodes derived from the model development data. ER-negative is indicated by *red lines*; ER-positive is indicated by *blue lines. ER* Oestrogen receptor

The breast cancer-specific mortality HR functions for age at diagnosis, tumour size and number of positive nodes are shown in Fig. 2. In ER-positive disease, after adjusting for other prognostic variables, there is an increase in risk of breast cancer-specific mortality in younger and older patients, with a substantial increase in risk for women diagnosed before the age of 35 years. The association between breast cancer-specific mortality and both tumour size and number of positive nodes was non-linear, with a more marked increase in risk with increasing size and increasing number of nodes in ER-positive disease. The corresponding baseline survival functions are given by the following equations:

$$ {\mathrm{BSb}}_{\mathrm{t}}\left(\mathrm{ER}\ \mathrm{negative}\right) = \hbox{-} 1.156 + 0.4707/{\mathrm{t}}^2\hbox{-}\ 3.514/\mathrm{t} $$

$$ {\mathrm{BSb}}_{\mathrm{t}}\left(\mathrm{ER}\ \mathrm{positive}\right) = 0.7424\ \hbox{-}\ 7.530/{\mathrm{t}}^{1/2}\hbox{-}\ 1.813* \ln \left(\mathrm{t}\right)/{\mathrm{t}}^{1/2} $$

**Fig. 3:**
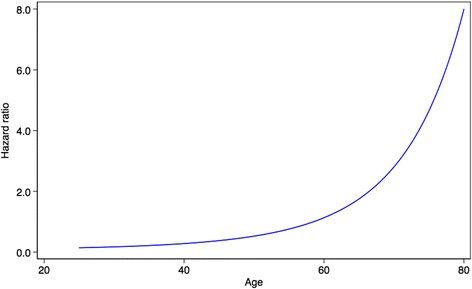
Age-specific HR for non-breast cancer mortality derived from the model development data. ER-negative is indicated by *red lines*; ER-positive is indicated by *blue lines. ER* Oestrogen receptor

The age-specific HRs for non-breast cancer mortality are shown in Fig. 3. The relevant baseline survival function is:

$$ {\mathrm{BSo}}_{\mathrm{t}}\left(\mathrm{non}\hbox{-} \mathrm{breast}\ \mathrm{mortality}\right) = \hbox{-} 6.053 + 1.080* \ln \left(\mathrm{t}\right) + 0.3255*{\mathrm{t}}^{1/2} $$

### Model calibration

The observed and predicted numbers of deaths from breast cancer and deaths from other causes are shown in Table 2. While there was no significant differences in the observed and predicted numbers of breast cancer deaths for the model fitting data, NTBCS or BCOS, the predicted number of breast cancer deaths was slightly over-estimated for POSH (*P* = 0.018). The number of predicted deaths from other causes was significantly lower than that observed for NTBCS (*P* = 0.039) and significantly higher for POSH (*P* < 0.001).

**Table 2:** Observed and predicted (PREDICT v2) deaths, by cause of death and data set

Study	Number	Observed	Predicted	Difference (%)	*P* value
Total mortality					
ECRIC	5738	1550	1600	3	0.21
BCOS	981	255	270	6	0.34
NTBCS	1944	488	468	−4	0.36
POSH	2609	544	621	12	0.00
Total	11,272	2837	2958	4	0.023
Breast cancer-specific mortality					
ECRIC	5738	932	953	2	0.48
BCOS	981	227	244	7	0.25
NTBCS	1944	325	331	2	0.74
POSH	2609	527	581	9	0.018
Total	11,272	2011	2110	5	0.027
Non-breast cancer mortality					
ECRIC	5738	618	646	4	0.25
BCOS	981	28	26	−9	0.66
NTBCS	1944	163	137	−19	0.039
POSH	2609	17	39	57	<0.001
Total	11,272	826	848	3	0.44

*Abbreviations: BCOS* Breast Cancer Outcome Study of Mutation Carriers, *ECRIC* Eastern Cancer Registration and Information Centre, NTBCS Nottingham/Tenovus Breast Cancer Study, POSH Prospective study of Outcomes in Sporadic and Hereditary breast cancer

The observed and predicted (v1 and v2) breast cancer deaths in the model development data set by age at diagnosis, tumour size, nodes positive and tumour grade are shown in Table 3. Overall, the calibration of PREDICT v1 and v2 was good for ER-negative disease (observed breast cancer deaths 333 compared with 326 predicted by PREDICT v1 and 330 by PREDICT v2). PREDICT v1 overestimated the number of breast cancer deaths in women with ER-positive breast cancer by 13% (599 observed compared with 677 predicted, *P* = 0.003). However, the number of breast cancer deaths in younger women with ER-positive disease was underestimated, whereas that in older women was overestimated. In contrast, the calibration of PREDICT v2 was very good for ER-positive disease (626 predicted, *P* = 0.27).

**Table 3:** Observed and predicted breast cancer specific mortality at ten years in model fitting data set by estrogen receptor status, age at diagnosis, tumour size, nodes positive and tumour grade

	Number cases	Number of deaths			PREDICT v1		PREDICT v2	
		PREDICT v1	PREDICT v2	Observed	Predicted − observed (%)	P-value	Predicted − observed (%)	P-value
Age at diagnosis								
ER negative								
20–29	11	3.6	3.7	4	-11	0.81	-7	0.88
30–39	105	32.5	34.5	40	-19	0.19	-14	0.35
40–49	210	60.8	66.1	60	1	0.92	10	0.45
50–59	304	88.7	94.7	88	1	0.94	8	0.49
60–69	200	62.8	64.6	63	0	0.98	3	0.84
70–79	190	77.9	67.4	78	0	0.99	-14	0.20
Total	1020	326.3	331.1	333	-2	0.71	-1	0.92
ER positive								
20–29	12	1.5	3.3	5	-70	0.004	-34	0.35
30–39	209	38.9	43.1	43	-10	0.51	0	0.99
40–49	765	108.9	112.3	107	2	0.86	5	0.62
50–59	1556	171.6	162.8	157	9	0.26	4	0.65
60–69	1214	157.2	142.5	139	13	0.15	3	0.77
70–79	962	198.6	162.4	148	34	0.0003	10	0.26
Total	4718	676.7	626.4	599	13	0.0028	5	0.27
Tumour size (mm)								
ER negative								
0–9	57	6.9	8.8	9	-23	0.43	-2	0.94
10–19	324	65.6	77.6	73	-10	0.36	6	0.60
20–29	337	102.3	105.5	108	-5	0.58	-2	0.81
30–49	218	95.3	86.6	90	6	0.58	-4	0.72
50+	84	56.0	52.5	53	6	0.69	-1	0.94
Total	1020	326.3	331.1	333	-2	0.71	-1	0.92
ER positive								
0–9	528	19.0	15.7	11	72	0.07	42	0.24
10–19	1976	145.7	149.0	129	13	0.17	15	0.10
20–29	1329	221.4	205.9	217	2	0.77	-5	0.44
30–49	665	194.8	168.1	161	21	0.015	4	0.58
50+	220	95.8	87.8	81	18	0.13	8	0.47
Total	4718	676.7	626.4	599	13	0.0028	5	0.27
Nodes positive								
ER negative								
0	536	103.7	101.6	107	-3	0.75	-5	0.59
1	150	41.7	48.5	49	-15	0.26	-1	0.95
2–4	168	71.1	76.8	68	5	0.71	13	0.31
5–9	92	53.7	53.5	57	-6	0.65	-6	0.63
10+	74	56.0	50.5	52	8	0.59	-3	0.84
Total	1020	326.3	331.1	333	-2	0.71	-1	0.92
ER positive								
0	2832	182.7	188.4	180	1	0.84	5	0.54
1	703	87.1	86.7	69	26	0.05	26	0.057
2–4	713	169.3	146.9	136	25	0.01	8	0.37
5–9	304	129.8	113.9	122	6	0.49	-7	0.45
10+	166	107.7	90.4	92	17	0.13	-2	0.87
Total	4718	676.7	626.4	599	13	0.0028	5	0.27
Grade								
ER negative								
1	27	4.0	4.9	2.9	38	0.58	70	0.36
2	240	62.8	69.4	75.8	-17	0.10	-8	0.44
3	753	259.5	256.7	235.8	10	0.14	9	0.19
Total	1020	326.3	331.1	314.5	4	0.51	5	0.36
ER positive								
1	963	34.2	36.2	30	14	0.47	21	0.30
2	2696	319.2	305.1	296	8	0.19	3	0.60
3	1059	323.3	285.0	273	18	0.0051	4	0.48
Total	4718	676.7	626.4	599	13	0.0028	5	0.27

Table 4 shows the observed and predicted (v1 and v2) breast cancer deaths in the combined validation data sets by ER status, age at diagnosis, tumour size, nodes positive and tumour grade. The results by individual data set and ER status for age at diagnosis, tumour size, nodes positive and tumour grade are shown in Additional file 1: Tables S1–S4. PREDICT v1 over-estimated the number of breast cancer deaths in ER-negative cases by 11% (446 observed compared with 492 predicted, *P* = 0.034). This over-estimation was most notable in the larger tumours and in the high-grade tumours. In contrast, the calibration of PREDICT v2 in ER-negative cases was better (predicted 480, *P* = 0.12). The calibration of both PREDICT v1 and PREDICT v2 was good in ER-positive cases (observed breast cancer deaths 633 compared with 643 [*P* = 0.67] and 677 [*P* = 0.09] predicted by v1 and v2, respectively). However, as previously described, PREDICT v1 under-estimated breast cancer-specific mortality in women diagnosed with ER-positive disease diagnosed under 50 years of age. In contrast, PREDICT v2 slightly over-predicted the number of breast cancer deaths in women diagnosed under the age of 30 years (48 predicted vs. 34 observed, *P* = 0.047). Both PREDICT v1 and v2 tended to under-estimate breast cancer mortality in women with small ER-positive tumours and over-estimate mortality in women with larger ER-positive tumours.

**Table 4:** Observed and predicted breast cancer specific mortality at ten years in combined validation data sets by estrogen receptor status, age at diagnosis, tumour size, nodes positive and tumour grade

	Number cases	Number of deaths			PREDICT v1		PREDICT v2	
		PREDICT v1	PREDICT v2	Observed	Predicted − observed (%)	P-value	Predicted − observed (%)	P-value
Age at diagnosis								
ER negative								
20–29	92	27.1	25.6	24	13	0.55	6	0.76
30–39	855	246.8	246.8	226	9	0.18	9	0.18
40–49	414	130.9	124.4	122	8	0.41	2	0.83
50–59	165	48.7	46.3	45	8	0.60	3	0.85
60–69	117	35.9	33.8	28	28	0.19	21	0.32
70–79	11	3.0	2.8	1	202	0.24	180	0.28
Total	1654	492.4	479.7	446	11	0.034	8	0.12
ER positive								
20–29	140	24.3	47.7	34	-28	0.050	40	0.047
30–39	1633	276.0	316.5	304	-9	0.092	4	0.48
40–49	1063	203.3	186.0	167	22	0.010	11	0.16
50–59	467	56.6	51.1	49	16	0.31	4	0.77
60–69	517	72.9	66.9	72	1	0.91	-7	0.53
70–79	55	9.9	8.8	7	41	0.36	26	0.54
Total	3875	643.0	677.1	633	2	0.67	7	0.09
Tumour size (mm)								
ER negative								
0–9	96	11.1	13.3	12	-7	0.79	10	0.73
10–19	559	108.9	118.9	110	-1	0.92	8	0.41
20–29	524	149.9	144.3	140	7	0.42	3	0.72
30–49	354	150.2	131.4	130	16	0.10	1	0.91
50+	121	72.9	71.8	54	35	0.027	33	0.04
Total	1654	493.0	479.7	446	11	0.034	8	0.12
ER positive								
0–9	352	20.2	17.5	27	-25	0.13	-35	0.024
10–19	1428	130.7	142.2	151	-13	0.076	-6	0.46
20–29	1111	188.6	195.5	192	-2	0.81	2	0.80
30–49	695	180.7	189.5	165	10	0.24	15	0.07
50+	289	123.3	132.3	98	26	0.023	35	0.00
Total	3875	643.7	677.1	633	2	0.67	7	0.09
Nodes positive								
ER negative								
0	937	180.2	165.3	167	8	0.33	-1	0.89
1	232	64.4	70.0	60	7	0.58	17	0.23
2–4	300	127.8	132.1	117	9	0.34	13	0.19
5–9	101	58.9	57.2	55	7	0.61	4	0.77
10+	84	61.7	55.1	47	31	0.062	17	0.28
Total	1654	493.0	479.7	446	11	0.034	8	0.12
ER positive								
0	2,085	169.0	190.6	188	-10	0.14	1	0.85
1	675	97.8	109.1	100	-2	0.83	9	0.39
2–4	734	187.8	187.5	181	4	0.62	4	0.63
5–9	245	109.6	109.5	94	17	0.14	17	0.14
10+	136	79.5	80.3	70	14	0.29	15	0.25
Total	3875	643.7	677.1	633	2	0.67	7	0.09
Grade								
ER negative								
1	44	6.2	7.3	7	-12	0.74	4	0.91
2	183	41.7	45.6	39	7	0.68	17	0.33
3	1427	445.1	426.8	400	11	0.033	7	0.19
Total	1654	493.0	479.7	446	11	0.034	8	0.12
ER positive								
1	658	29.4	31.4	27	9	0.66	16	0.43
2	1730	212.1	230.8	219	-3	0.64	5	0.44
3	1487	402.2	414.9	387	4	0.45	7	0.17
Total	3875	643.7	677.1	633	2	0.67	7	0.09

### Model discrimination

The PREDICT model discrimination by data set is shown in Table 5. The AUC in the model-fitting data was similar for PREDICT v1 and v2 for ER-negative disease (0.724 and 0.726, *P* = 0.67), whereas the AUC was slightly smaller for v1 than v2 in ER-positive disease (0.791 and 0.796, *P* = 0.028). The AUC values for PREDICT v1 and v2 were similar in the individual validation sets for both ER-negative and ER-positive disease, although in the combined validation data, PREDICT v2 performed slightly better for ER-positive disease than for ER-negative disease (AUC 0.760 vs. 0.750, *P* = 0.016).

**Table 5:** Comparison of discrimination (AUCs) of PREDICT v1 and PREDICT v2, by data set and oestrogen receptor status

Study	ER status	Predict v1	Predict v2	*P* value
ECRIC	Negative	0.724	0.726	0.67
ECRIC	Positive	0.791	0.796	0.028
ECRIC	All	0.801	0.805	0.014
BCOS	Negative	0.650	0.632	0.87
BCOS	Positive	0.737	0.741	0.52
BCOS	All	0.737	0.734	0.45
NTBCS	Negative	0.671	0.680	0.32
NTBCS	Positive	0.787	0.790	0.57
NTBCS	All	0.770	0.772	0.63
POSH	Negative	0.717	0.715	0.76
POSH	Positive	0.741	0.746	0.36
POSH	All	0.735	0.741	0.22
Combined validation	Negative	0.698	0.696	0.70
Combined validation	Positive	0.750	0.760	0.016
Combined validation	All	0.747	0.752	0.058

*Abbreviations: BCOS* Breast Cancer Outcome Study of Mutation Carriers, *ECRIC* Eastern Cancer Registration and Information Centre, NTBCS Nottingham/Tenovus Breast Cancer Study, POSH Prospective study of Outcomes in Sporadic and Hereditary breast cancer

### Goodness of fit

**Fig. 4:**
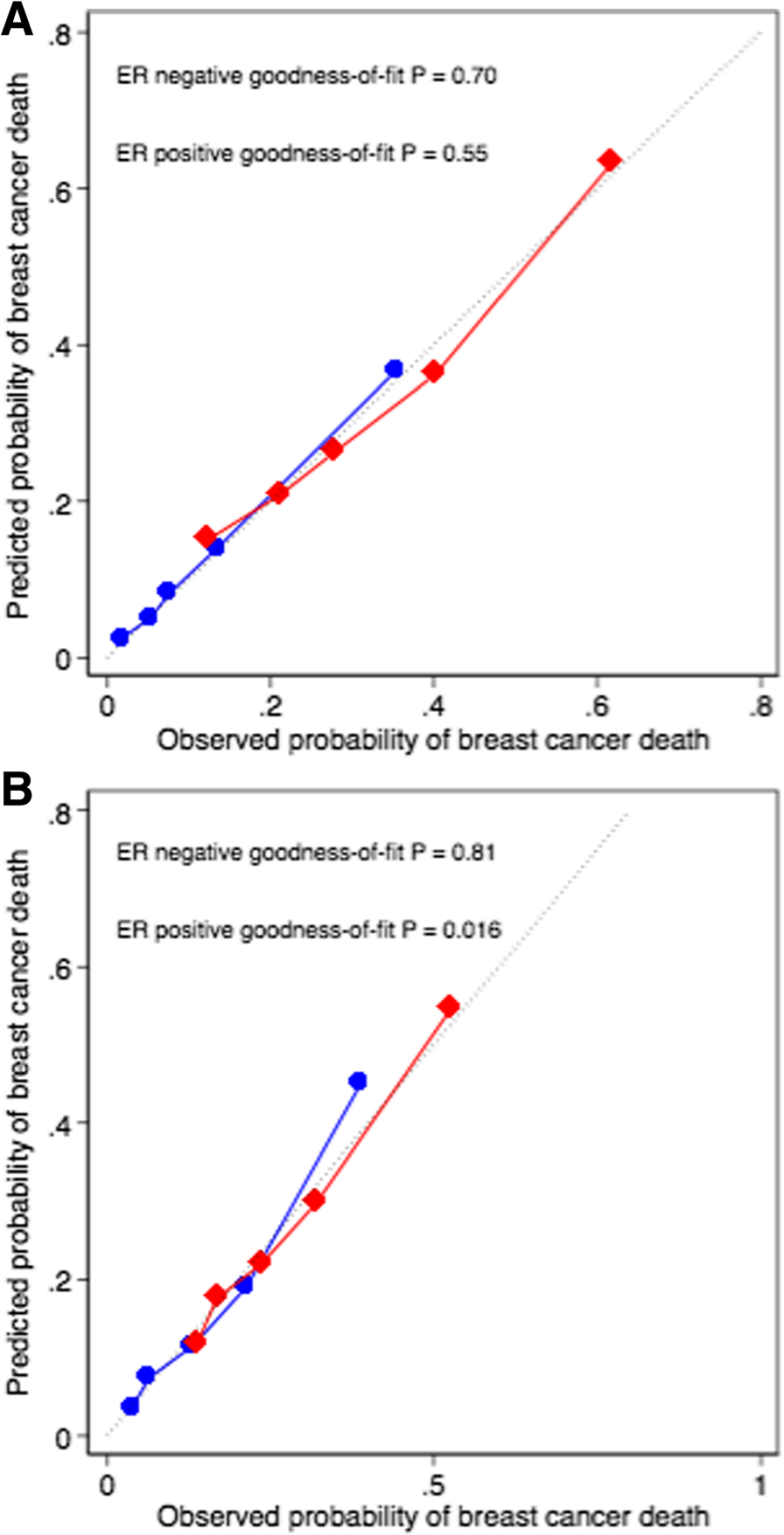
Observed and predicted breast cancer deaths at 10 years by quintile of predicted risk. **a** Model development data. **b** Validation data. ER-negative is indicated by *red lines*; ER-positive is indicated by *blue lines. ER* Oestrogen receptor

The observed and predicted breast cancer deaths by quintile of predicted risk for PREDICT v2 are shown in Fig. 4a for the model development data and in Fig. 4b for the validation data. The observed values differed significantly from the predicted for the ER-positive cases in the validation data (χ^2^ = 13.2, 5 *df*, *P* = 0.020), with a slight over-estimation in the highest risk quintile (325 deaths predicted vs. 293 observed).

## Discussion

We have refitted the prognostic model underlying the PREDICT breast cancer prognostication and treatment benefit tool using the original data used to develop the model with updated survival time data and using a sophisticated approach to modelling the data with multivariable fractional polynomial models in a Cox regression framework. The association between tumour size and node status with prognosis is, of course, well-established, but the difference in the shape of the non-linear associations in ER-positive and ER-negative disease has not previously been described. Similarly, multiple studies have reported an association of young age at diagnosis with a poor prognosis (e.g., [17–21]), but those studies that have used multivariable models have simply adjusted for ER status and as a result have not reported the notable difference in the age-specific relative hazards between ER-positive and ER-negative disease demonstrated by our analysis.

The calibration of the new model is better than that of the original model for breast cancer-specific mortality in the model development data set. In three independent validation data sets, the calibration of PREDICT v1 and v2 is similar, with v1 being slightly better for ER-positive disease and v2 being slightly better for ER-negative disease. There was little difference in the discrimination of PREDCIT v2 compared with v1 for ER-negative disease, but for ER-positive disease, v2 performed slightly better in both model-fitting and validation data sets.

Prediction of non-breast cancer deaths was excellent in the model development data set but not as good in the validation data sets. The under-prediction of non-breast cancer deaths in the NTBCS data set is likely to be partly due to the fact that this is a cohort diagnosed in the 1980s, when population death rates were higher than at the time the model development cohort was ascertained. Non-breast cancer mortality was also under-predicted in the BCOS case series, which was ascertained in the 1990s, although this under-prediction was not significant. Other mortality was significantly over-predicted in the POSH case series. Because this case series is almost contemporaneous with the model development cases, differences in population mortality rates are unlikely to be the explanation. However, the participation of eligible women in POSH is liable to be subject to a healthy cohort bias, with women with better general health being more likely to participate than those with poorer general health.

The PREDICT model was originally developed using data from patients treated in the United Kingdom between 1999 and 2003. Since then, there have been several advances in breast cancer treatment, including the introduction of sentinel node biopsy, intensity-modulated radiotherapy, targeted therapies such as trastuzumab, and taxane-based (third-generation) chemotherapy. As a result, the original model has been updated to include the prognostic effect of HER2 status and the benefit of trastuzumab. Although the majority of the model development cohort who received adjuvant chemotherapy were treated with second-generation regimens, the POSH validation cohort was diagnosed and treated during 2000–2007, and many were treated using taxane-based adjuvant chemotherapy.

## Conclusions

In an era of precision oncology, accurate, well-validated models that predict patient outcomes are invaluable clinical tools. We have derived an improved version of the PREDICT prognostication and treatment benefit model to reduce some of the limitations of the original model. The new model has been validated in three independent data sets and performs well. It has been implemented online and will continue to aid clinical decision making in clinical practice.

## Additional file

Additional file 1: The observed and predicted (PREDICT v1 and v2) events by individual data set and ER status for age at diagnosis (**Table S1**), tumour size (**Table S2**), nodes positive (**Table S3**) and tumour grade (**Table S4**). (XLSX 35 kb)

## Abbreviations

BCOS: Breast Cancer Outcome Study of Mutation Carriers
ECRIC: Eastern Cancer Registration and Information Centre
ER: Oestrogen receptor
HER2: Human epidermal growth factor receptor 2
MI: Mortality index
NTBCS: Nottingham/Tenovus Breast Cancer Study
PI: Prognostic index
POSH: Prospective study of Outcomes in Sporadic and Hereditary breast cancer
